# Effects of leadership style on coach-athlete relationship, athletes’ motivations, and athlete satisfaction

**DOI:** 10.3389/fpsyg.2022.1012953

**Published:** 2022-12-12

**Authors:** Hao Jin, Seungmo Kim, Adam Love, Yun Jin, Jie Zhao

**Affiliations:** ^1^FIBA China, Beijing, China; ^2^Department of Sport and Physical Education, Faculty of Social Sciences, Hong Kong Baptist University, Kowloon, Hong Kong SAR, China; ^3^Department of Kinesiology, Recreation, and Sport Studies, College of Education, Health, and Human Sciences, The University of Tennessee, Knoxville, Knoxville, TN, United States; ^4^College of Physical Education, Hebei Normal University, Shijiazhuang, Hebei, China

**Keywords:** democratic leadership, autocratic leadership, coach-athlete relationship, motivation, athlete satisfaction

## Abstract

**Introduction:**

The current study investigated the impacts of autocratic and democratic leadership styles on the coach-athlete relationship, athletes’ motivations, and athlete satisfaction.

**Methods:**

Survey data were collected from 298 student-athletes (male = 157; 52.7%, female = 141; 47.3%) from 20 different Chinese collegiate sports. The Structural Equation Model was used to test the hypothesized model.

**Results:**

The results indicated democratic leadership had a direct positive influence on the coach-athlete relationship, while autocratic leadership had no direct effect. Both leadership styles did not influence autonomous motivation. The coach-athlete relationship, meanwhile, had a full mediation effect between democratic leadership and athletes’ motivation and satisfaction. In addition, autonomous motivation had a partial mediation effect between the coach-athlete relationship and athlete satisfaction.

**Discussion:**

Ultimately, the findings of the current study underscore the need for coaches and administrators to understand the impact of different leadership styles and highlight the importance of democratic leadership in improving athletes’ psychological outcomes.

## Introduction

A variety of different leadership styles are widely used among leaders in business, sports, and politics (Farh and Cheng, 2000). Leading a team of athletes entails unique complexities compared to other business and organizational contexts, and there has been an increased interest in research about the effectiveness of coaching leadership styles in recent years (Castillo and Espinosa, 2014; Jowett, 2017). A successful sports team will need coaches to properly guide their athletes to maintain healthy relationships with fellow team members while also fostering a high level of performance (Mallett, 2005). Numerous studies have found that coaches’ leadership behaviors can play a crucial role in athletes’ psychological development and satisfaction (Weiss and Friedrichs, 1986), training efficiency and game outcomes (Becker and Wrisberg, 2008), and team cohesion (Jowett and Chaundy, 2004; Vincer and Loughead, 2010).

The Multidimensional Model of Leadership in Sport (MML) developed by Chelladurai and Saleh (1980) based on the unique demands of sport is a pioneering model of sports leadership. The model has been frequently discussed and explored by sport scholars (Hampson and Jowett, 2014; Chia et al., 2015). The MML delineates three states of leadership behavior—required, actual, and preferred. Behaviors related to adhering to government regulations, conference compliance, and institutional structure are defined as *required*. Behavior based on the coach’s ability and philosophy, as well as other situational factors, reflect the *actual* leadership state. Last, *preferred* leadership behavior refers to meeting the goals and needs of athletes (Dupuis et al., 2006). The degree of consistency of the three leadership behavior states is an indicator of team performance and athlete satisfaction, which is associated with coaching effectiveness (Chia et al., 2015). According to the MML, to become an effective coach, one most adapt and adopt appropriate coaching styles in different situations. For instance, a coach who leads high school level teams may need to employ different coaching styles depending on the team’s goals (e.g., league championship or individual skill development), the familiarity and maturity of the players, and the schedule of a weekly training regimen.

Although the autocratic leadership style may achieve successful results in some cases, athletes’ ability level (e.g., mental development) and team conditions must be considered when coaches employ different leadership styles. Because autocratic leadership reduces the opportunity for athletes’ internal decision-making, it should be matched with a level of development from athletes to follow coaches’ commands and instructions. In other words, to achieve the functional purpose of different leadership styles (i.e., autocratic or democratic) an appropriate level of ability by athletes to follow leadership is crucial to avoid conflicts (Yang and Jowett, 2010). For instance, if a coach seeks to exert assertive control over athletes, those who prefer a higher level of autonomy may feel and tense or strained relationship with the coach. On the other hand, imbalance may also occur when the coach tends to be democratic, yet some athletes prefer clear guidance from coaches and want instructions that create a highly structured setting with specific plans and goals. Such disparity can lead to dissatisfaction and poor performance among athletes on a team. As a result, different leadership styles may have an important impact in sports teams, and coaches must think carefully in selecting and implementing leadership behaviors.

A “centralized sports governance” system has been used in China to organize elite sports, including recruiting youth athletes and hosting major events (Yang et al., 2015). Unlike the primarily free-market system of the US, the Chinese government maintains control of funding, training, and operations for the sport performance sector. Under this government-led system, leadership studies in China have largely focused on the relationships between leadership behavior and team performance and cohesion (Cui, 2010; Li et al., 2017) rather than athletes’ psychological well-being. China’s centralized sports institutions and cultural background may encourage the adoption of autocratic leadership styles, as performance is valued above and beyond other interests, such as social development and health. For example, paternalistic leadership based on the cultural roots of confucianism tends to emphasize strict discipline and authority. The three components of paternalistic leadership are authoritarianism, benevolence, and moral leadership (Farh and Cheng, 2000). In fact, some studies have focused on paternalistic leadership to analyze the leadership style of Chinese coaches given the cultural background factors (Li and Li, 2021; Li et al., 2021).

Overall, there is a lack of coaching leadership research that explores the unique sport culture in China, which has a strong focus on performance and elite sport. Many young Chinese athletes may spend more time with their coaches than with their parents, making it particularly important to understand the impact of coaches, not only with respect to advancing athletes’ sports skills, but also influencing their education and holistic development (Zhu et al., 2017). Hence, the current study was designed to investigate the influence of leadership behaviors, including both autocratic and democratic leadership, to enrich the theoretical framework and increase the range of understanding about coaching in Chinese universities. The current study can help practitioners better understand effective teaching methods in practice by examining the impact of coaches’ leadership. Most significantly, the study may assist coaches in choosing appropriate leadership styles to interact with their athletes throughout training and competition to improve their performance and holistic development. Therefore, the main purpose of the current study was to investigate the impacts of autocratic and democratic leadership styles on the coach-athlete relationship, athletes’ motivations, and athlete satisfaction in Chinese collegiate athletics.

## Theoretical foundations

**FIGURE 1:**
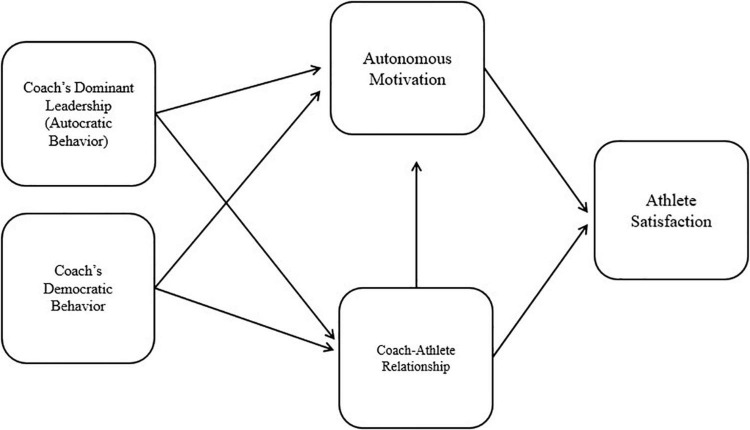
Proposed research model.

As shown in Figure 1, the conceptual model of the current study includes four constructs: (a) leadership style (i.e., autocratic leadership and democratic leadership), (b) coach-athlete relationship (CAR), (c) autonomous motivation, and (d) athlete satisfaction.

### Leadership in sport

The success of a sports team can depend on a coach’s leadership style, and research has identified several theories to determine the most effective coaching approaches (Jowett, 2017). In particular, the comparative effectiveness of democratic and autocratic coaching styles has been a frequent topic of investigation (Case, 1984). Many theories of situational leadership were developed in the 1960s and 1970s, which include contingency theory (Fiedler, 1967), path-goal theory (House, 1971), and the situational leadership model (Hersey and Blanchard, 1982). A key principle of situational leadership in sport is the leader’s ability to adapt to the needs and situations of athletes (Kim et al., 2021). Since athletes may experience ups and downs in their skill development and psychological growth, coaches need to think carefully about the overall dynamic relationship and find the most effective patterns with the athletes they mentor. Given that situational leadership does not involve only a single skill or approach, it can be difficult for coaches to master a diverse set of coaching behaviors for a variety of situations.

One of the most influential leadership frameworks in sport has been Chelladurai and Saleh’s (1980) MML, which was established based on the interactive behaviors of sports group members. To measure leadership styles within the MML, Chelladurai and Sarah developed the Leadership Scale for Sports (LSS). The LSS organized athletes’ perceived leadership style into five major categories: training and instruction, democratic behavior, autocratic behavior, social support, and positive feedback. Training and instruction referred to the essential roles of a coach in improving the performance level of athletes. Democratic behavior, meanwhile, reflected the extent to which the coach allows participation by athletes in the decision-making process. The third factor, autocratic behavior, indicated that “a coach keeps apart from the athletes and stresses his or her authority in dealing with them” (Chelladurai and Saleh, 1980, p. 41). Social support is evident when the coach is involved in satisfying the interpersonal needs of athletes. Last, positive feedback refers to the coach expressing appreciation and complimenting the athletes for their performance and contributions.

As oppositional decision-making styles, autocratic and democratic leadership approaches each have distinct advantages and limitations (Chelladurai and Saleh, 1980). Autocratic behavior, one of the most demanding leadership styles, emphasizes the coach’s authority over athletes in sports environments. With autocratic leadership, the dominance of the coach’s decision-making and personal power may limit athletes’ freedom of action but also facilitates quick problem-solving, which is often necessary for competitive sport environments. Autocratic leadership behaviors may increase athletes’ focus, which could increase practice effectiveness in some situations (Yang and Jowett, 2010). For example, Castillo and Espinosa (2014) found that individuals who were in the process of learning to master a dance skill were significantly impacted by autocratic teaching techniques. They discovered that, despite the limitations of the authoritarian approach, it set clear goals for achievement to lessen students’ loss of concentration during the learning process, which in turn led to a more efficient improvement in performance. However, due to the lack of encouragement associated with autocratic coaching behavior, autocratic leaders often impede athlete’s motivation from the psychological level, which may negatively impact the coach-athlete relationship (Mallett, 2005). Autocratic coaching style may also have a negative impact on athletes’ intrinsic motivation and feelings of relatedness (Hollembeak and Amorose, 2005). A higher level of autocratic behavior by coaches is associated with athletes who report higher levels of anxiety and burnout with lower levels of enjoyment and perceived competence (Price and Weiss, 2000). Overall, while autocratic leadership styles may have positive effects with respect to skill acquisition and performance in some situations, they also tend to negatively impact athletes in important ways.

In a contrast with an autocratic approach, Gastil (1994) made an important distinction between leadership and authority. In particular, he defined democratic leadership as performing three functions: distributing responsibility, empowering, and aiding deliberation (Gastil, 1994). In sports, implementing democratic leadership behaviors may entail the head coach sharing authority with a team captain or position group leader, allowing them to take some responsibility for the team’s progress and skill development. Under democratic leadership styles, athletes have more power to decide how they train and compete (Cruz and Kim, 2017). Higher levels of democratic leadership have been linked to more positive and less negative psychological outcomes for athletes (Price and Weiss, 2000). However, highly democratic approaches may also engender conflicts with athletes as well as disrespect and disobedience (Foels et al., 2000). A coach’s choices about the extent to which to employ democratic or autocratic leadership behavior may be influenced by factors such as gender (Wałach-Biśta, 2019), competition level, and sports type (e.g., team sports or individual sports). For instance, Terry and Howe (1984) discovered that coaching effectiveness was highly correlated with task dependence in the sport, and team sports that require a high level of teamwork and interaction (e.g., basketball) favored more authoritarian leadership styles.

Literature regarding situational leadership theory generally supports the idea that the coach should be flexible and adopt different leadership styles dependent upon the context. The adaptability of leaders is a critical principle in situational leadership in sports and has been identified as a key component of coaching mastery (Kim et al., 2021). Coaches in a setting such as collegiate sport must work with players who have varied levels of understanding due to their distinct educational backgrounds, athletic ambitions, and training experiences. Such factors make it challenging for coaches to maintain positive coach-athlete relationships and athlete satisfaction. Given the complexity involved in situational leadership, further research is required to understand the relationship between various leadership styles and important outcomes in sport, such as the coach-athlete relationship and athlete satisfaction.

### Outcomes of leadership style

#### Coach-athlete relationship

Athletes have interpersonal relationships with a variety of people in sport, including teammates, parents, coaches, and staff members. Given the fact that young athletes may spend more time with coaches than their parents in some elite sport contexts, the relationship quality with the coach is highly important, directly affecting the athletes’ skill development and competitive performance (Jowett, 2017). Jowett and Poczwardowski (2007) defined the coach-athlete relationship as “a situation in which a coach’s and an athlete’s cognitions, feelings, and behaviors are mutually and causally interconnected” (p. 4).

Examining behavioral, affective, and cognitive elements involved in leadership are important to understanding social behavior, such as the coach-athlete relationship (Jowett and Ntoumanis, 2004). Investigating the dynamic nature of CAR is essential for strengthening coaching effectiveness and fostering optimal physical and psychological performance of athletes (Jowett, 2017). Research regarding the influence of relationship quality indicates that CAR can impact important outcomes, such as moral disengagement (Chen et al., 2016) and social environment (Jowett, 2007). In fact, athletes’ relationships with coaches may have a significant impact on psychological outcomes and long-term stability, effecting not only their sport performance, but also their holistic development (Kim et al., 2020).

Given the important influence of a coach, it is essential to understand how different leadership styles may affect the relationships between coaches and athletes. In the context of sports in China, Zhu et al. (2017) found that the authoritarian behavior of coaches was the factor that athletes perceived as most detrimental to team effectiveness. Gao et al. (2021) similarly discovered that autocratic behavior appeared to have substantial detrimental impacts on athlete engagement and CAR. Relatively few studies, however, have investigated the connection between different leadership styles and the coach-athlete relationship (Jowett and Chaundy, 2004; Hampson and Jowett, 2014), particularly in the context of East Asia.

#### Motivation

Motivation has been extensively studied as a crucial factor influencing athletic success (Vallerand, 2007). Understanding and promoting athlete motivation has been demonstrated to have a significant influence on athletes’ performance (Mallett, 2005), cognition (Ryan and Deci, 2000), and behaviors (Li et al., 2021). Due to the importance of motivation in sports performance, much research has investigated the ways in which coaches’ behaviors, such as decision-making style, reward distribution, and feedback methods, are essential factors affecting athletes’ motivation (Mageau and Vallerand, 2003).

The two most well-known theories of motivation in sports psychology are self-determination theory (SDT; Deci and Ryan, 1985; Ryan and Deci, 2000) and achievement goal theory (AGT; Nicholls, 1989). SDT focuses on intrinsically motivated psychological behavior based on three basic needs—competence, relatedness, and autonomy (Deci and Ryan, 1985). On the other hand, AGT places more emphasis on goal orientations, particularly task and ego orientation (Nicholls, 1989). The principles of intrinsic and extrinsic motivation are important areas of focus within SDT. The model expands on the three fundamental requirements (i.e., competence, relatedness, and autonomy) to form a continuous and unified structure that can locate and illuminate various factors impacting athletes’ motivation (Spray et al., 2006).

Deci and Ryan (1985) developed a motivation continuum that segmented motivation into six components. From highest to lowest, the levels of self-determination were labeled intrinsic, integrated, identified, introjected, external, and amotivation. The term intrinsic motivation (IM) referred to instances in which an activity is done for inherent reasons. Extrinsic motivation (EM), meanwhile, included a group of motivations with varying degrees of autonomy—integrated, identified, introjected, and external. Last, amotivation (AM) indicated a lack of autonomy. In sports, an athlete’s actions may reveal their motivation. For instance, athletes who feel satisfaction in the sport or find their value by participating in sport are more likely to be motivated by internal factors. In contrast, engaging in sport to escape punishment and guilt or seek praise and approval from others tends to be influenced by extrinsic motivations, which reflect non-autonomous intentions (Amorose and Anderson-Butcher, 2007).

Existing research evaluating IM and EM to determine the quality of motivational orientations has identified the differentiation between autonomous and controlled motivation as being important (Ratelle et al., 2007). Whereas autonomous behavior is typically self-initiated, controlled motivation occurs when an action results from external influence. To calculate controlled motivation, researchers have often used the mean score of external and introjected motivation (Sheldon and Elliot, 1998). Conversely, investigators have identified autonomous motivation as a mix of intrinsic and identified motivation (Fenton et al., 2014). Koestner et al. (2008) found that increasing autonomous motivation was more successful than decreasing controlled motivation when examining the relative role of the two in the achievement of personal goals.

Coaches’ decision-making styles, autocratic or democratic, can have substantial implications with respect to athletes’ motivations. Creating an autonomy-supportive motivational climate has been found to serve an essential role in supporting athletes to develop strong commitment and interest in sports (Mallett, 2005). Hollembeak and Amorose (2005) established that autocratic and democratic styles were the two behaviors that had a substantial indirect effect on autonomy in all five categories of leadership styles under the LSS (Chelladurai and Saleh, 1980). Democratic leader behavior has received positive feedback from athletes at all levels, including elementary (Fenton et al., 2014), secondary (Spray et al., 2006), high school (Amorose and Anderson-Butcher, 2007), club teams (Vincer and Loughead, 2010), and college. Autonomy supportive coaching behaviors, such as encouragement, may have significant benefits in boosting intrinsic motivation and engagement of athletes (Hollembeak and Amorose, 2005). In contrast, autocratic behavior has been found to hinder athletes’ initiative (Hollembeak and Amorose, 2005).

The motivational model of the coach-athlete relationship, presented by Mageau and Vallerand (2003), illustrated the positive effect of coaches’ autonomy supportive behavior on CAR and motivation of athletes. Wu et al. (2014) found that authoritarian behavior was negatively related to autonomy, relatedness, and intrinsic motivation, whereas democratic behavior had the opposite impact on these outcomes in Chinese collegiate sports. Consequently, to improve coaching effectiveness in China, it may be useful to advance the autonomous motivation of athletes as a key to developing effective coaching behavior and healthy coach-athlete relationships.

#### Athlete satisfaction

Athletes’ satisfaction has been observed as a key reflection of many coaching characteristics, including coaches’ personality (Yang et al., 2015), physical behaviors (Davis et al., 2019), and leadership style (Kim et al., 2020). Therefore, considering athletes’ satisfaction is an important practice for coaches to achieve successful performance and training efficiency by valuing the effect of different leadership styles. Riemer and Toon (2001) revealed that an athlete’s ability level affected their preference for types of leadership behavior and level of satisfaction. Weiss and Friedrichs (1986), meanwhile, found that the democratic leadership style positively impacted satisfaction among college athletes. Many studies on the coach-athlete relationship have found associations among CAR, motivation, and satisfaction (Lorimer and Jowett, 2009). Multiple studies (Koestner et al., 2008; Grant and Berg, 2011) have shown that autonomous motivation, controlled motivation, and satisfaction interacted with each other. Jowett (2017), meanwhile, asserted that CAR was central to coaching effectiveness, and its quality greatly impacted athletes’ levels of satisfaction, pleasure, and wellbeing. Davis et al. (2019) similarly found relationships between the quality of CAR and athletes’ experiences of sport satisfaction.

In the past decade, research on coaches’ leadership styles in China has concentrated on the connection between team cohesion and team effectiveness for college athletes (Cui, 2010; Chen et al., 2016; Li et al., 2017; Zhu et al., 2017; Gao et al., 2021). Thus, the current study attempted to fill a gap in Chinese coaches’ research on the impact of autocratic and democratic leadership styles among Chinese coaches on athletes’ satisfaction levels and enhancing coaching efficiency through CAR and motivation. Specifically, it was hypothesized that CAR and motivation would mediate the association between coaching style and satisfaction. Overall, the following hypotheses were proposed based on the aforementioned research background:

Hypothesis 1: Leadership style (H1a: autocratic leadership, H1b: democratic leadership) will influence coach-athlete relationship.

Hypothesis 2: Leadership style (H2a: autocratic leadership, H2b: democratic leadership) will influence autonomous motivation.

Hypothesis 3: Coach-athlete relationship will mediate the relationship between leadership style (H3a: autocratic leadership, H3b: democratic leadership) and autonomous motivation.

Hypothesis 4: Coach-athlete relationship will mediate the relationship between leadership style (H4a: autocratic leadership, H4b: democratic leadership) and athlete satisfaction.

Hypothesis 5: Autonomous motivation will mediate the relationship between leadership style (H5a: autocratic leadership, H5b: democratic leadership) and athlete satisfaction.

Hypothesis 6: Autonomous motivation will mediate the relationship between coach-athlete relationship and athlete satisfaction.

## Materials and methods

### Participation and survey procedure

The population of the current study was composed of current college athletes in China. An online survey with a convenience sampling method was used to collect data. The researchers sent an initial WeChat message with a link to the survey to athletes from a variety of sports at the collegiate level in China to recruit participants. The data collection period was from April 22 to May 1, 2022. In addition to inviting them to complete the questionnaire, recipients were also asked to forward the link to other student-athletes in their networks. Two follow-up e-mail reminders were sent to encourage participation. A total of 157 male (52.7%) and 141 female (47.3%) athletes from 20 different sports, including volleyball (*n* = 109; 36.6%), basketball (*n* = 54; 18.1%), track and field (*n* = 39; 13.1%), football (*n* = 33; 11.1%), table tennis (*n* = 13; 4.4%), and other sports completed the questionnaire. The majority of participants were first-class athletes (*n* = 138; 46.3%) and second-class athletes (*n* = 138; 46.3%) according to the Chinese Athletes Technical Classification Standard (General Administration of Sport of China, 2010). Participants were primarily between 16 and 24 years old (*n* = 276; 92.6%), and the majority (*n* = 207; 69.5%) had trained for between 5 and 10 years in their sport. More demographic information is shown in Table 1.

**TABLE 1:** Demographic profile of participants (*n* = 298).

		*n*	%
Gender	Male	157	52.7
	Female	141	47.3
Age	16–19	65	21.8
	20–24	211	70.8
	25–29	17	5.7
	Over 40	3	1.0
Sports	Volleyball	109	36.6
	Basketball	54	18.1
	Track and field	39	13.1
	Football	33	11.1
	Table tennis	13	4.7
	Swimming	11	3.7
	Badminton	8	2.7
	Taekwondo	7	2.3
	Others	24	8.1
Level of competition	International	1	0.3
	National	21	7.0
	Level 1	138	46.3
	Level 2	138	46.3
	Less than 5 years	32	10.7
Career experience	5–10 years	207	69.5
	11–15 years	48	16.1
	Over 15 years	11	3.7

### Instruments

Forty-one items from previously validated scales were used to measure autocratic coaching style (five items), democratic coaching style (five items), coach-athlete relationship (eleven items), motivation (nine items), athlete satisfaction (five items) and demographic information. The 10 items measuring autocratic and democratic coaching behaviors were adopted from the LSS (Chelladurai and Saleh, 1980). Each item began with the statement “my coach…” and included items such as “refuses to compromise on a point” and “speaks in a manner not to be questioned” for autocratic behavior and “let his/her athletes share in decision making” and “encourages athletes to make suggestions on conducting practices” for democratic behavior. These items were scored using 7-point Likert-type scales ranging from 1 (strongly disagree) to 7 (strongly agree). To measure an athlete’s perception regarding the relationship with his/her coach, Coach-Athlete Relationship Questionnaire (CART-Q; Jowett and Ntoumanis, 2004) was used. The scale included 11 items, such as “I appreciate the sacrifices my coach has experienced in order to improve performance,” “I am committed to my coach,” and “I am ready to do my best.” The items were also scored using a 7-point Likert-type scale ranging from 1 (strongly disagree) to 7 (strongly agree). Nine items from the Sport Motivation Scale (SMS-II), developed by Pelletier et al. (2013) were utilized to examine autonomous motivation. The items included “Because it gives me pleasure to learn more about my sport” and “Because participating in sport is an integral part of my life.” Athlete satisfaction was measured using job satisfaction scales developed by Judge et al. (1998), modified to be used in the context of sports. The items included “I feel fairly satisfied with my team” and “Each day at practice seems like it will never end (reversed coded).”

In developing the questionnaire, the survey was initially written in English because the scales of the four concepts that the current research adopted were originally developed in English. The English version was then translated into Chinese, as all the participants in the current study were native Chinese speakers. The translation was conducted by two individuals with a graduate degree in business and communication data science who were familiar with organizational behavior literature and fluent in English and Mandarin. The Chinese version was then back-translated into English by another individual, who was a Ph.D. candidate in sports psychology with similar language qualifications to the previous translators. Finally, 10 athletes in China were recruited for a pilot study to assess the survey’s ease of use and clarity.

### Data analyses

The current study sequentially conducted a confirmatory factor analysis (CFA) to evaluate the measurement model and structural equation model (SEM) analysis to examine the research model based on Anderson and Gerbing’s (1988) two-step approach using AMOS 27. For both the CFA and SEM, the present study used indexes [i.e., chi-square, the Steiger-Lind Root Mean Square Error of Approximation (RMSEA), Standardized Root Mean Squared Residual (SRMR), Comparative Fit Index (CFI), and Tucker-Lewis fit index (TLI)] to assess an overall fit of structure since the indexes are often recommended to evaluate structural equation models (Browne and Cudeck, 1992; Hu and Bentler, 1999; Kline, 2015). In addition, Cronbach’s α coefficients were calculated to verify the internal consistency of each measurement scale’s components. Descriptive statistics were compiled to provide relevant demographic information about the sample as well as the means and standard deviations of each construct. Next, SEM was used to test the proposed model. The bootstrapping method was used to test the mediating effects of the proposed model, which may provide additional implications for leadership style, CAR, autonomous motivation, and athlete satisfaction. The research model also was assessed by the same indexes previously used for the CFA.

## Results

### Measurement model

The results of an initial CFA showed unacceptable model fit [Chi-square statistic = 2,089.045, df = 550, CFI = 0.863, TLI = 0.852, RMSEA = 0.097, and SRMR = 0.068] since TLI and CFI should be equal to or greater than 0.9 (Hair et al., 2010) and RMSEA and SRMR should be equal to or less than 0.08 (Tabachnick et al., 2007) to be acceptable. Further, Parsimonious Fit Indices (PNFI and PCFI) were 0.761 and 0.798, respectively. Thus, the investigators removed four items (two for autocratic coaching style and two for athletic satisfaction) due to low factor loadings (below 0.4). The removed items were “My coach works relatively independent of the athletes” and “My coach does not explain his/her action” for autocratic leadership, as well as “Each day at practice seems like it will never end (Reversed Code)” and “I consider my team and my sport rather unpleasant (Reversed Code)” for athlete satisfaction. Since negatively phrased items may be associated with respondent errors (Sonderen et al., 2013), reversed codes may have contributed to the low factor loadings in. After dropping those items, the results of the CFA indicated an acceptable fit for the measurement model [Chi-square statistic = 1,199.304, df = 414, CFI = 0.927, TLI = 0.918, RMSEA = 0.080, and SRMR = 0.060] with Parsimonious Fit Indices (PNFI and PCFI) of 0.796 and 0.826, respectively. Convergent validity of the measures was established because construct reliability (CR) and average variance extracted (AVE) were greater than 0.7 and 0.5, respectively (Ahmad et al., 2016). Discriminant validity was also established since correlation coefficients among latent variables were smaller than the square roots of AVEs. The results of convergent and discriminant validity along with the results of the correlation analysis are shown in Tables 2, 3. In terms of internal consistency, Cronbach’s α for autocratic leadership, democratic leadership, coaching-athlete relationship, motivation, and athlete satisfaction were 0.715, 0.818, 0.864, 0.882, and 0.904, respectively (Lance et al., 2006).

**TABLE 2:** Measurement model.

	λ	AVE	CR	α
**Autocratic leadership**		0.512	0.749	0.760
My coach refuses to compromise on a point	0.671			
My coach keeps to himself/herself	0.799			
My coach speaks in a manner not to be questioned	0.668			
**Democratic leadership**		0.700	0.832	0.906
My coach asks for the opinions of athletes on strategies for specific competitions	0.883			
My coach lets his/her athletes share in decision-making	0.839			
My coach encourages athletes to make suggestions on conducting practices	0.871			
My coach lets the group set its own goal	0.778			
My coach lets the athletes try their own way, even if they make mistakes	0.708			
**Coach-athlete relationship**		0.751	0.916	0.970
I like my coach/My coach likes me	0.926			
I trust my coach/My coach trusts me	0.911			
I respect my coach/My coach respects me	0.867			
I appreciate the sacrifices my coach has experienced in order to improve performance/My coach appreciates the sacrifices I have experienced to improve my performance	0.875			
I am close to my coach/My coach is close to me	0.859			
I am committed to my coach/My coach is committed to me	0.914			
I feel that my sports career is promising with my coach/My coach believes that his/her coaching career is promising with me	0.867			
I am ready to do my best/My coach is ready to do his/her best	0.864			
I am at ease/My coach is at ease	0.756			
I am responsive to his/her efforts/My coach is responsive to my efforts	0.863			
I adopt a friendly stance/My coach adopts a friendly stance	0.820			
**Autonomous motivation**		0.778	0.900	0.971
Because it gives me pleasure to learn more about my sport	0.846			
Because I find it enjoyable to discover new performance strategies	0.849			
Because it is very interesting to learn how I can improve	0.876			
Because practicing sports reflects the essence of whom I am	0.850			
Because participating in sport is an integral part of my life	0.874			
Because through sport, I am living in line with my deepest principles	0.904			
Because I have chosen this sport as a way to develop myself	0.930			
Because I found it is a good way to develop aspects of myself that I value	0.922			
Because it is one of the best ways I have chosen to develop other aspects of myself	0.885			
**Athlete satisfaction**		0.817	0.750	0.931
Most days I am enthusiastic about my work	0.917			
I feel satisfied with my present job	0.872			
I find real enjoyment in my work	0.909			

**TABLE 3:** Discriminant validity.

	1	2	3	4	5
(1) Autocratic leadership	(0.715)				
(2) Democratic leadership	−0.261**	(0.818)			
(3) CAR	−0.286**	0.676**	(0.867)		
(4) AM	−0.173*	0.573**	0.803**	(0.882)	
(5) AS	−0.212*	0.581**	0.829**	0.815**	(0.904)

Values on the diagonal denote square root of the AVEs. CAR, coach-athlete relationship; AM, autonomous motivation; AS, athlete satisfaction.

***p < 0.001; **p < 0.01; *p < 0.05.

### Descriptive statistics

Table 4 shows the means and standard deviations of each construct in terms of the gender of the sport and the sample as a whole. These data revealed that male and female athletes showed similar outcomes. For example, all the means for each construct were above the mid-point of the scale (4.00), with the exception of autocratic leadership. The means from both men and women were below the midpoint of the scale for autocratic leadership. Regarding the outcomes of coach leadership style, the means for the outcome variables ranged from 5.94 (athlete satisfaction) to 6.65 (coach-athlete relationship). The levels of all outcomes approached or exceeded 6 out of 7, which indicates athletes perceive a good relationship with their coaches and are satisfied with their current athletic careers overall.

**TABLE 4:** Descriptive statistics.

Variables			Gender			
	Overall		Male		Female	
	*M*	*SD*	*M*	*SD*	*M*	*SD*
Autocratic leadership	3.82	1.51	3.76	1.53	3.87	1.50
Democratic leadership	5.57	1.37	5.56	1.38	5.59	1.35
Coach-athlete relationship	6.65	1.41	6.62	1.52	6.67	1.26
Autonomous motivation	6.03	1.23	6.06	1.24	5.99	1.27
Athlete satisfaction	5.94	1.35	5.98	1.34	5.90	1.37

### Structural model

**FIGURE 2:**
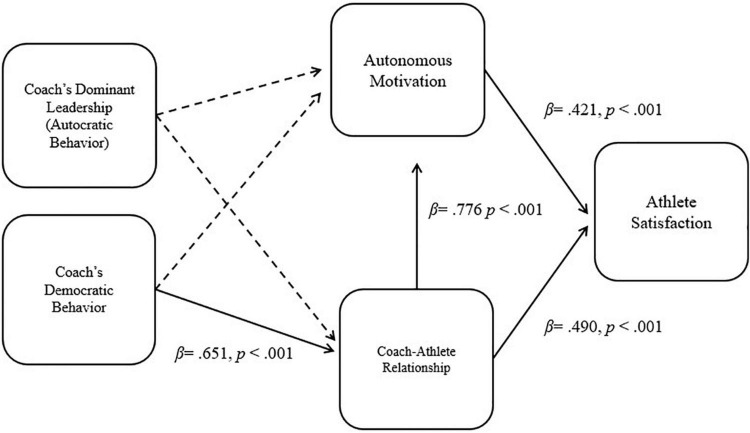
Proposed structured research model with path coefficients.

The structural model consisting of autocratic leadership, democratic leadership, CAR, and athlete satisfaction was tested using the maximum likelihood estimation method. The model fits of the revised structural model were acceptable [Chi-square statistic = 1,242.421, df = 446, CFI = 0.927, TLI = 0.919, RMSEA = 0.079, and SRMR = 0.059] with Parsimonious Fit Indices (PNFI and PCFI) of 0.801 and 0.834, respectively. Figure 2 shows the results of the revised structural model. The results indicated that autocratic leadership had no direct effect on the coach-athlete relationship, which rejected hypothesis 1a, whereas democratic leadership had a direct positive influence on coach-athlete relationship (β = 0.651, SE = 0.073, *p* < 0.001), which supported Hypothesis 1b. However, both leadership styles did not influence autonomous motivation, which rejected Hypothesis 2a and 2b.

The mediating effects to examine the internal mechanism among the variables in the proposed model were tested using the bootstrapping method; 5,000 bootstrap samples were generated using random sampling with replacement from actual data. Although the proposed model consisted of seven indirect paths to test the hypotheses, we tested only three direct paths because several direct paths to the proposed mediators were not supported. With respect to the mediation effects of ç between democratic leadership and outcomes (autonomous motivation and athlete satisfaction), the mediation effects were significant (Democratic leadership → CAR → autonomous motivation (β = 0.505, SE = 0.064, *p* < 0.001), Democratic leadership → CAR → athlete satisfaction (β = 0.558, SE = 0.054, *p* < 0.001). Therefore, the coach-athlete relationship had a full mediation effect between only democratic leadership and the outcome variables, which supported Hypothesis 3b and 4b but rejected Hypotheses 3a, 4a, 5a, and 5b. In addition, the mediation effects of autonomous motivation between CAR and athlete satisfaction showed a partial mediation effect with the indirect effect (CAR → autonomous motivation → athlete satisfaction: β = 0.327, SE = 0.075, *p* < 0.001) and the direct effect (CAR → athlete satisfaction: β = 0.490, SE = 0.097, *p* < 0.001). Therefore, Hypothesis 6 was rejected. Table 5 provides detailed results with respect to the direct and indirect effects.

**TABLE 5:** Direct and indirect effects using bootstrapping method.

Direct effect	β	SE	t	90% CI
Autocratic leadership → CAR	−0.098	0.065	−1.508	−0.205, −0.002
Autocratic leadership → AM	0.057	0.034	1.676	−0.008, 0.123
Democratic leadership → CAR	0.651	0.073	8.918	0.584, 0.730
Democratic leadership → AM	0.064	0.078	0.821	−0.081, 0.205
CAR → AM	0.776	0.088	8.818	0.659, 0.886
AM → AS	0.421	0.092	4.576	0.288, 0.561
CAR → AS	0.490	0.097	5.052	0.348, 0.617
**Indirect effect of CAR**				
Democratic leadership → AM	0.505	0.064	7.891	0.409, 0.621
Democratic leadership → AS	0.558	0.054	10.333	0.469, 0.645
**Indirect effect of AM**				
CAR → AS	0.327	0.075	4.360	0.219, 0.471

CAR, coach-athlete relationship; AM, autonomous motivation; AS, athlete satisfaction.

## Discussion

This study aimed to discover the impacts of autocratic and democratic leadership style along with democratic leadership on coach-athlete relationships, autonomous motivation, and athletes’ satisfaction in Chinese collegiate athletics. A total of eleven hypotheses were developed based on the MML (Chelladurai and Saleh, 1980) and associated research on the relationships between coaches and athletes. Among the proposed hypotheses, three hypotheses were supported. The findings of this study help provide a more comprehensive understanding of the impact of coaching style on the coach-athlete relationship and athletes’ motivation and satisfaction in Chinese collegiate athletics.

The descriptive statistics of this study illustrated that collegiate athletes in China reported average mean scores of 3.82 for autocratic leadership, 5.57 for democratic leadership, 6.65 for coach-athlete relationships, 6.03 for autonomous motivation, and 5.94 for athlete satisfaction (on a 7-point scale). The average mean scores of male and female athletes were comparable, implying that gender has less influence on the perception of coaching leadership style in this context. A trend had been apparent in prior research in that athletes in Western countries perceived low levels of autocratic leadership among their coaches (Jowett and Chaundy, 2004; Hampson and Jowett, 2014), whereas studies in East Asian countries, such as China (Cui, 2010) and Korea (Cruz and Kim, 2017), found higher levels of authoritarian leadership among coaches. The results of the current study, however, revealed that athletes’ perceived levels of autocratic leadership among coaches to be below the midpoint of the scale. Due to selections for Chinese national teams being typically made from among athletes in the professional team system, coaches in collegiate sports may be under less pressure to perform and utilize less authoritarianism compared to those at the professional and elite levels. On the other hand, the average score for democratic leadership was 5.57, higher than that found in other studies in East Asia, suggesting that democratic leadership may be becoming more regularly applied in the Chinese university context. Given the high scores of CAR (6.65), autonomous motivation (6.03), and satisfaction (5.94), the findings highlight important relationships between different coaching styles and these three variables.

### Academic implications

There are three key findings to highlight from the SEM analysis in this study. First, with respect to hypothesis 1a and 2a, the effects of autocratic leadership on CAR and autonomous motivation were found to be insignificant in this study. These results are notable because prior research has frequently found autonomous motivation to have a direct or indirect relationship with autocratic coaching styles. Hollembeak and Amorose (2005), for example, found that authoritarian and democratic styles had significant indirect effects on autonomy, while Wu et al. (2014) study on the motivation of Chinese college athletes determined that authoritarian behavior had a significant negative relationship with autonomy and intrinsic motivation. However, the results of the current study showed that the autocratic leadership style did not have a significant relationship with autonomous motivation. Grant and Berg (2011) suggested that since the two motivations, autonomous and controlled, often coexist, examining the simultaneous action of both motivations is worthwhile. The fact that autocratic leadership only had a minimal effect on autonomous motivation might be explained by the coexistence and ambivalence of different types of motivation. For example, the nature of the coach-athlete relationship varies depending on different cultural norms in different countries (Yang et al., 2015), and respect for those in positions of authority (i.e., coaches) is considered a traditional norm in Chinese culture. However, athletes’ respect for a coach may develop differently and be affected by varying factors in different (e.g., Western) cultural contexts. Culture norms for respecting authority figures may have influenced the sensitivity of Chinese college athletes toward autocratic behaviors, which in turn led to a non-significant relationship between CAR and autonomous motivation (Lee, 2017). Furthermore, the findings revealed no link between autocratic leadership and CAR, either positive or negative, similar to the results of Li and Li (2021) in a study among Chinese youth soccer players.

Secondly, with respect to hypothesis 1b, the democratic coaching approach directly and positively impacted the quality of relationships between coaches and athletes. Moreover, with respect to hypothesis 2b, democratic leadership had an indirect influence on autonomous motivation and athlete satisfaction through CAR. These results are largely in line with previous research findings on such relationships. Through more democratic leadership behaviors, coaches appear to build trust and a sense of respect with their athletes (Gao et al., 2021). Mageau and Vallerand (2003), p. 886 identified specific behaviors that contributed to the autonomy-supportive climate, including “providing choice to their athletes within specific limits and rules” and “providing the opportunity for athletes to take initiative and act independently.” In addition, Mageau and Valler and highlighted how the autonomy-supportive conduct of coaches improved the quality of CAR and boosted players’ motivation. The results of the current study confirm the mediating role of CAR in the association between democratic leadership style and autonomous motivation and satisfaction. In alignment with Jowett (2017), CAR appears to be at the core of coach effectiveness. The outcomes of the current study (i.e., CAR, autonomous motivation, and athletes’ satisfaction) have been frequently recognized as important influences for enhancing performance in sports psychology (Vallerand and Losier, 1999; Jowett, 2017). Therefore, the current study provides further support to the findings of prior studies regarding the effects of autocratic and democratic coaching styles (Jowett and Chaundy, 2004) by confirming that democratic leadership behaviors had a more positive influence on athletes’ psychological outcomes in Chinese collegiate athletics than autocratic leadership behaviors.

Finally, with respect to hypothesis 6, the current study discovered a partial mediating effect of autonomous motivation between CAR and athlete satisfaction, confirming the association between these three variables observed in previous studies (Koestner et al., 2008; Grant and Berg, 2011). The results demonstrated that a number of criteria, including the quality of CAR and the athlete’s internal motivation, can be used to explain athlete satisfaction. Previous results had demonstrated that coaches employing democratic coaching behavior and encouraging athletes to make decisions for themselves improve team cohesion and overall satisfaction (Weiss and Friedrichs, 1986). Athletes who feel trusted and have a strong emotional attachment with their coaches tend to show increased positive motivation and encouragement of feedback from their teammates (Watson and Kleinert, 2019). Ultimately, fostering autonomous motivation among athletes appears to be an important area on which coaches should focus their attention.

### Practical implications

The current study’s findings supported the hypotheses that different leadership coaching approaches, particularly democratic leadership, can affect athletes’ satisfaction levels, interpersonal relationships, and motivation. It is crucial for coaches and college sports team administrators to thoroughly understand the ways in which different coaching styles may increase the quality of connections with players and affect their behaviors. The findings of the current study demonstrated that by maintaining a good relationship with the coach and having a high level of autonomous motivation, athletes’ higher levels of satisfaction could be vital to their performance (Weiss and Friedrichs, 1986). The situational leadership model emphasizes the importance of coaches’ flexibility in applying different leadership techniques in accordance with athletes’ needs and goals (Hersey and Blanchard, 1982). Since the autocratic leadership style did not significantly impact athletes’ relationships and autonomous motivation in the current study, Chinese athletes may have a high tolerance for autocratic behaviors due to cultural influences. While Chinese college coaches have the discretion to use an authoritarian approach to achieve efficient results in the preseason or during short-term intensified training, relying solely on autocratic actions would be unlikely to improve the quality of relationships or increase autonomous motivation, despite the fact that China has a “centralized sports governance” system (Yang et al., 2015), and paternalistic leadership is a common leadership style in Chinese culture (Wu et al., 2014). Conversely, coaches should involve the athletes in preparing training plans and developing strategies in competition to avoid monotony and repetition in offseason training. Democratic behaviors that coaches adopt, when appropriate, can make athletes feel respected and trustworthy and satisfy their psychological needs. In responding to the coaches’ effort and care, college athletes would be likely to show more initiative by cultivating healthy relationships with their coaches in response to democratic approaches.

The results of the current study confirmed that both the quality of the coach-athlete relationship and autonomous motivation had a significant positive impact on athlete satisfaction, which may deliver an important message to a sports team. Respect for and obedience toward coaches and other authority figures has been a traditional component of Chinese culture. Athletes, as subordinates, have tended to obey coaches’ demands and refrain from expressing their true feelings to a coach. The conventional view of coaches as authoritarian team leaders has also prevented them from developing the practice of encouraging communication (Lee, 2017). Therefore, it is critical for coaches and team managers to maintain effective two-way communication. The connection and confidence between coaches and athletes should be boosted and supervised to maintain a long-term healthy relationship (Gao et al., 2021). Coaches, as leaders, should regularly and effectively seek to understand athletes’ emotional and psychological changes to evaluate their status and interpersonal relationships. At the same time, the managers of sports teams should foster an environment in which athletes have the opportunity to express their feelings and thoughts to the coach freely. Additionally, maintaining open lines of communication makes it easier for the coach to select the best coaching approach during practice and competition based on the status of the athletes and the team.

### Limitations and future research directions

Although this study contributes to the literature regarding the impacts of various leadership styles on Chinese college athletes, there are some important limitations. First, the current study only examined the influences of coach leadership styles based on the perspective of athletes. Price and Weiss (2000) explain that different leadership styles may also contribute to coaches’ burnout, which affects the coach-athlete relationship as well as the team’s long-term performance. In addition, coaches’ leadership style preferences are not immutable (Hersey and Blanchard, 1982), and the decision-making process for coaches differs from athletes’ considerations and motivation. At the same time, coaches’ perspectives can help further explain the interactive relationship when analyzing the connection between athletes and coaches. Additionally, the data for this study were collected through online surveys. Compared with face-to-face methods, the number of unqualified questionnaires through the online collection is greater (Heerwegh, 2009), further demonstrating the value of multiple methodological approaches when investigating this topic.

Although the current study adds to the base of information on Chinese coaching styles by analyzing the relationship between motivation and satisfaction among CAR, further investigation is required to more comprehensively identify additional factors that impact training effectiveness. The current study emphasized the impact of leadership style, particularly democratic leadership, on athlete outcomes in Chinese collegiate athletics. Of course, the authoritarian leadership style may also be useful in some contexts, such as with novice athletes who wish to improve their skills quickly (Castillo and Espinosa, 2014). Hypothetically, if Chinese youth athletes generally accept an authoritarian leadership style, they may prefer a less stressful or inexperienced democratic leadership style when competing in college. Building from the current study, a more comprehensive sample of athletes, including high school and youth athletes, can provide insight into satisfaction with different coaching leadership styles at different stages of development. Such insight may assist coaches in choosing appropriate leadership styles in different situations at various stages of athletes’ development in order to enhance athletes’ satisfaction and performance.

Finally, the current study is one of relatively few investigations into leadership style and its effect on athletes’ psychological outcomes in China. Such research in the context of China is particularly important, given that many young Chinese athletes may spend more time with their coaches than with their parents due to the high-stakes nature of sport in the country, affecting their sports skills, education, and holistic development (Zhu et al., 2017). While the bulk of research on coaching leadership has been conducted in Western nations, differing cultural norms may impact the nature of the coach-athlete relationship in different national contexts (Yang et al., 2015). In turn, additional cross-cultural research that compares leadership style, CAR, and related outcomes in East Asia and other regions will provide a valuable contribution to the field.

## Data availability statement

The raw data supporting the conclusions of this article will be made available by the authors, without undue reservation.

## Ethics statement

The studies involving human participants were reviewed and approved by Research Ethics Committee from Hong Kong Baptist University. Written informed consent to participate in this study was provided by the participants’ legal guardian/next of kin.

## Author contributions

HJ designed the computational framework and got involved in every part of the research. SK helped to develop the framework, analyzed the data, and wrote the results. AL helped to write the “Introduction” and “Discussion” of this research. YJ and JZ were in charge of data collection and also helped to write the literature reviews. All authors contributed to the article and approved the submitted version.
